# Acute effects of bench press conditioning performed with different velocity-loss thresholds on punch performance in elite adolescent boxers

**DOI:** 10.3389/fphys.2026.1973551

**Published:** 2026-10-14

**Authors:** Penglin Diao, Jinxuan Bao, Xiaoyu Liu, Zhaoqian Li, Yunsong Liu, Xundian Liu, Yayi Jiang, Yinkai Zhang, Ziyu Wang

**Affiliations:** 1 School of Sports Training, Wuhan Sports University, Wuhan, China; 2 School of Strength and Conditioning Training, Beijing Sport University, Beijing, China; 3 School of Arts and Sciences, Fuyao University of Science and Technology, Fujian, Fuzhou, China; 4 School of Physical Education, Shandong University, Shandong, Jinan, China; 5 Beijing Navigation School, Beijing, China; 6 Postgraduate Department, Belarusian State University of Physical Culture, Minsk, Belarus; 7 China Wushu School, Beijing Sport University, Beijing, China; 8 Faculty of Health, University of Canterbury, Christchurch, New Zealand

**Keywords:** adolescent athletes, boxing, combat sports, post-activation performance enhancement, punch performance, velocity loss, velocity-based training

## Abstract

**Background:**

Velocity-loss (VL) thresholds may help regulate the balance between potentiation and fatigue during post-activation performance enhancement protocols; however, their effects on boxing-specific performance remain unclear. This study investigated the acute effects of bench press conditioning regulated by different VL thresholds on straight-punch performance in adolescent boxers.

**Methods:**

Eighteen male boxers (17.6 ± 0.7 years) completed four conditions in a randomized, counterbalanced crossover design: an active control condition (CON; two sets of 10 bench press repetitions using a habitual warm-up load below 30% one-repetition maximum [1RM]) and three VL conditions consisting of two sets of bench press at 85% 1RM terminated at 10% (VL10%), 20% (VL20%), or 30% (VL30%) velocity loss. Lead- and rear-hand peak punch velocity (PPV), peak punch force (PPF), and an estimated peak punch-power index (PPP) were assessed at baseline and 4, 8, 12, and 16 min after conditioning. Outcomes were analyzed using linear mixed-effects models, followed by Dunnett-adjusted comparisons of each VL condition with CON. Additional Tukey-adjusted pairwise comparisons were performed among VL10%, VL20%, and VL30%.

**Results:**

Significant condition × time interactions were observed for rear-hand PPV, lead- and rear-hand PPF, and lead- and rear-hand PPP (all *p* ≤ 0.046), but not for lead-hand PPV (*p* = 0.391). At 8 and 12 min, VL10% and VL20% produced higher rear-hand PPV, PPF, and PPP and higher lead-hand PPP than CON (*p* ≤ 0.039; Hedges’ g = 0.835–1.267). In contrast, VL30% produced lower rear-hand PPV, lead- and rear-hand PPF, and rear-hand PPP than CON at 4 min and remained lower for selected outcomes at 8–12 min (*p* ≤ 0.046; g = −2.439 to −0.814). Direct VL comparisons showed no significant differences between VL10% and VL20%, whereas both conditions produced higher values than VL30% for several outcomes at selected time points (*p* ≤ 0.045). Most between-condition differences were no longer evident at 16 min.

**Conclusion:**

Under the present protocol in the 18 male adolescent boxers studied, VL10% and VL20% were associated with favorable differences in several punch-performance outcomes after 8–12 min of recovery, whereas VL30% was associated with lower performance during the earlier recovery period. Direct comparisons showed no significant differences between VL10% and VL20%, whereas both conditions produced higher values than VL30% for several outcomes at selected time points. However, because repetition volume was not matched, these findings do not isolate the effect of VL threshold or establish an optimal threshold.

## 1 Introduction

Post-activation performance enhancement (PAPE) refers to an acute improvement in physical performance following short-duration, high-intensity or near-maximal intensity muscle contractions (Tillin and Bishop, 2009). This enhancement is primarily manifested as transient increases in strength, speed, or power (Wilson et al., 2013). This effect is believed to be closely associated with increased neuromuscular excitability, potentially involving improved motor unit recruitment efficiency and enhanced Ca^2+^ sensitivity of muscle fibers (Hodgson et al., 2005). Previous studies indicate that PAPE is determined by the balance between potentiation and fatigue induced by the conditioning activity (Blazevich and Babault, 2019). Performance enhancement is more likely to be observed when potentiation outweighs residual fatigue, resulting in a transient improvement in subsequent performance (Seitz and Haff, 2016). Therefore, a key challenge in PAPE research is to induce sufficient neuromuscular activation while minimizing fatigue (Finlay et al., 2022).

Based on these mechanisms, PAPE has been widely applied in sports dominated by explosive performance (Şener et al., 2025). It is commonly used as a warm-up or contrast training strategy to enhance subsequent explosive performance (Gouvêa et al., 2013; Wilson et al., 2013). In highly trained boxers, a PAPE protocol involving a 10-s cycling sprint increased mean power and total work during a subsequent 30-s Wingate cycling test compared with a control condition; however, that study did not assess punch performance (Zhang et al., 2024). In recent years, the PAPE effect in upper-body explosive training has received increasing attention. Recent research in amateur boxers has shown that PAPE, alone or combined with caffeine, can enhance upper-body bar velocity during the bench press (Wang et al., 2026). Systematic reviews and meta-analyses have further indicated that conditioning activities can also induce small magnitude performance enhancement in upper-body ballistic exercises such as the bench press throw (Krzysztofik et al., 2021). However, these analyses also indicate that existing upper-body PAPE research has primarily examined non-specific performance outcomes such as the bench press throw (Krzysztofik et al., 2021; Finlay et al., 2022), which may not fully reflect the force-transfer demands of boxing-specific punching actions (Liu et al., 2022). For combat sports centered on punching speed and explosive power (e.g., boxing), punching performance depends on the efficient transfer of force through the lower-limb–trunk–upper-limb kinetic chain (Piorkowski et al., 2011; Deng et al., 2025). Cui et al. reported time-specific improvements in punch performance following squat conditioning regulated by velocity-loss thresholds in boxers (Cui et al., 2024). Although the bench press primarily involves upper-body pressing and does not reproduce the complete punching action (Dinu and Louis, 2020; Liu et al., 2022), bench-press bar velocity at 80% 1RM has been associated with rear-hand punch velocity in professional boxers (López-Laval et al., 2020). This association provides a rationale for testing bench-press conditioning, but its acute effects on punching must be assessed using boxing-specific outcomes.

Velocity loss (VL) reflects the decline in bar velocity within a set and can be used to monitor within-set fatigue and determine when to end the set (González-Badillo et al., 2017; Pareja-Blanco et al., 2017). In the present study, all three VL conditions used the same relative bench-press load (85% 1RM), and the assigned VL threshold (10%, 20%, or 30%) determined when each set ended and how many repetitions were completed. In this context, evidence from upper-body conditioning is particularly relevant to this protocol. Tsoukos et al. tested bench press at 40% and 60% 1RM with 10% or 30% VL in resistance-trained men. At the group level, bench-press throw velocity increased 4–12 min after the 60% 1RM/10% VL protocol, but not after the other tested protocols (Tsoukos et al., 2019). Together, these studies leave unclear whether VL-regulated bench press at 85% 1RM affects punching in adolescent boxers, whose responses may also be influenced by biological maturation (Behm, 2018; Tumkur Anil Kumar et al., 2021).

Therefore, the purpose of this study was to examine the magnitude and time course of straight-punch performance responses following bench press conditioning regulated by different velocity loss thresholds (10%, 20%, and 30% VL) relative to an active control condition involving low-load bench press exercise in adolescent boxers. Punch force and punch velocity were assessed under each condition, and performance responses were tracked from 4 to 16 min following the conditioning activity. Based on the premise that lower VL may induce less fatigue and allow potentiation to predominate, it was hypothesized that (1) lower-VL conditions would show more favorable time-specific differences relative to CON than the higher-VL condition; and (2) favorable performance differences would be most apparent approximately 8–12 min following the conditioning activity. Potential differences in the response patterns of the lead- and rear-hand straight punches were additionally explored descriptively. This study aimed to further the understanding of VL-regulated PAPE and provide practical guidance for boxing-specific training, particularly in adolescent athletes.

## 2 Methods

### 2.1 Experimental design

This study employed a randomized, counterbalanced crossover experimental design, in which each participant completed four bench-press conditions in a counterbalanced order: (a) a control condition (CON), and three velocity-based training (VBT) conditions in which bench-press sets were terminated at velocity loss (VL) thresholds of (b) 10% (VL10%), (c) 20% (VL20%), and (d) 30% (VL30%) (Jukic et al., 2023). The order of the four conditions was determined using a computer-generated counterbalanced randomization sequence prepared before data collection. In each condition, straight punch performance was assessed at five standardized time points: at baseline (T0) and at 4 min (T1), 8 min (T2), 12 min (T3), and 16 min (T4) following the assigned condition (Cui et al., 2024). A washout period of 72 hours was implemented between each condition to ensure complete recovery and to mitigate interference from residual training effects. All sessions were conducted at the same time of day to control for circadian effects. Participants maintained their regular boxing training throughout the study period but were required to avoid any high-intensity upper-body training for at least 24 hours prior to each test. Additionally, they were instructed to abstain from caffeine, alcohol and to maintain an identical diet for 24 hours before each test. All testing procedures were performed in the same indoor facility under controlled environmental conditions (room temperature 22-24 °C, relative humidity 40-60%). To reduce potential bias, participants were not informed of the prespecified VL threshold assigned to each session; however, complete participant blinding was not possible because differences in repetition number and set duration could be perceived. Intervention staff were necessarily aware of the assigned condition because they monitored repetition velocity and terminated each set according to the prescribed VL threshold. Statistical analyses were performed with condition codes concealed from the data analyst (**Figure 1**).

**Figure 1:**
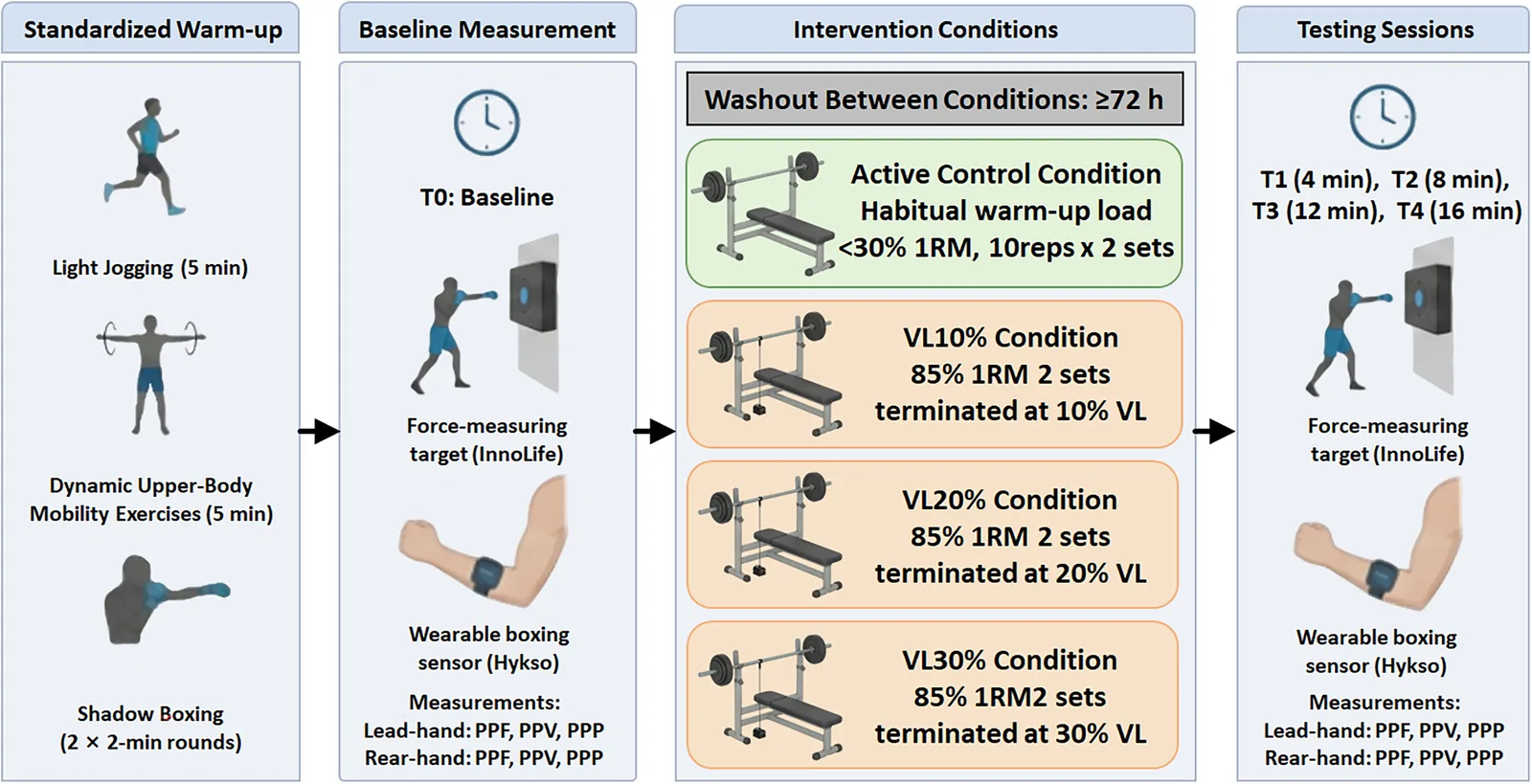
Experimental design. Overview of the randomized, counterbalanced crossover experimental conditions (CON, VL10%, VL20%, VL30%) and the timing of punch-performance assessments (T0–T4). Bench press conditioning was performed at 85% 1RM for VL conditions, with sets in the VL conditions terminated at predefined velocity-loss thresholds, and punch outcomes (PPF, PPV, PPP) were quantified using force-target and wearable-sensor measurements. Created by the authors using Microsoft PowerPoint for Microsoft 365, Version 2408.

### 2.2 Participants

An *a priori* sample-size calculation was conducted using G*Power 3.1 (Heinrich-Heine-Universität Düsseldorf, Düsseldorf, Germany) using a repeated-measures ANOVA framework as an approximate basis for recruitment (α = 0.05, power = 0.80, effect size f = 0.30, correlation among repeated measures = 0.50, nonsphericity correction ϵ = 1.0). The calculation indicated that a minimum of 15 participants was required. Because the primary analysis involved a four-condition × five-time-point crossover design analyzed using linear mixed-effects models, the G*Power calculation was regarded as an approximate recruitment calculation rather than an exact power analysis of the condition × time interaction in the final model. To allow for potential attrition or unusable data, we aimed to enroll 18 participants. Twenty-one male boxers from the Shandong Provincial Boxing Team were screened for eligibility. Three were excluded: one because of an injury within the three months before testing and two because their bench-press 1RM did not exceed their body mass. The remaining 18 were enrolled in the study. All participants held a national second-class athlete certificate in boxing under China’s athlete grading system and had achieved a top-three placing in a provincial-level boxing competition. All athletes were right-handed. Participants were aged 16–19 years (17.6 ± 0.7 years), including seven participants younger than 18 years. They had 3.2 ± 1.7 years of systematic boxing training experience and completed 27.8 ± 5.2 h of boxing training per week. Inclusion criteria were: i) bench press 1RM greater than body weight (Folhes et al., 2022); ii) proficiency in the straight punch technique; and iii) no injuries within the three months prior to testing. Exclusion criteria included: i) cardiovascular or neuromuscular diseases; ii) recent use of medications affecting neuromuscular function; or iii) a history of upper-limb surgery or severe injury within the past six months. Participants aged 18 years or older provided written informed consent before participation. Participants younger than 18 years provided written assent, and written informed consent was obtained from a parent or legal guardian before enrollment. The study protocol was approved by the Ethics Committee of Beijing Sport University (Approval No. 2024322H) and conducted in accordance with the Declaration of Helsinki (**Table 1**).

**Table 1:** Participant characteristics (Mean ± SD).

Variables	Mean ± SD
Age (years)	17.6 ± 0.7
Height (cm)	179.5 ± 5.2
Body mass (kg)	67.2 ± 7.1
1RM (kg)	85.6 ± 9.8
1RM/body mass	1.3 ± 0.3
Boxing experience (in years)	3.2 ± 1.7
Boxing training volume per week (hours)	27.8 ± 5.2

### 2.3 Experimental procedures

All tests were conducted in a controlled laboratory environment. One week prior to the formal experiment, participants completed a familiarization session, which included the determination of bench press 1RM, technical standardization of the bench press, adaptation to velocity monitoring equipment, and practice of the straight punch testing protocol. 1RM determination followed the recommendations of the National Strength and Conditioning Association (NSCA) (Haff and Triplett, 2021). Specifically, subjects completed an incremental load protocol comprising three attempts at 40%, three at 70%, and one at 90% of their self-reported 1RM. Thereafter, the load was increased by 0.5 to 2.5 kg after each successful attempt until no further weight could be lifted. Participants were instructed and supervised to minimize learning effects and reinforce technical consistency across testing sessions. All participants performed the punch tests using their habitual boxing stance. Lead-hand and rear-hand punches were defined according to each athlete’s habitual stance rather than handedness alone.

In the formal experiment, participants first performed a standardized warm-up (5 min light jogging, 5 min dynamic upper-body mobility exercises, and two 2 min rounds of shadow boxing). Across all bench press conditions, the technical execution was standardized. Participants used a medium grip width and performed each repetition with a controlled eccentric phase (approximately 2 seconds), without pausing at the bottom position. This no-pause technique was selected to avoid introducing an isometric hold into the conditioning stimulus and was consistent with procedures used in previous bench-press research (Wilk et al., 2019). Concentric movement was performed with maximal intended velocity, with no pause at lockout. All sessions were supervised to ensure consistent technique. In the control condition, participants completed an active upper-body protocol using the same bench press movement rather than a passive control condition. Participants were instructed to select a light load that they habitually used for bench press warm-up, with the selected load required to remain below 30% of their individual 1RM. They completed two sets of 10 repetitions with a 1-min rest interval between sets. This protocol provided an active comparison involving the same general movement pattern and warm-up exposure. Exact participant-level external loads used during CON were not retained; therefore, the mean absolute load, mean relative load, and volume-load for this condition could not be calculated retrospectively. In the VL conditions, participants performed the bench press at a prescribed load of 85% 1RM as the pre-activation stimulus, with the corresponding external load rounded to the nearest 2.5 kg. A linear position transducer (GymAware RS; Kinetic Performance Technologies, Canberra, Australia) was used to monitor the mean concentric velocity (MCV) of each repetition in real time. Linear position transducers have been shown to provide valid and reliable velocity measurements during resistance training exercises, making them appropriate for real-time monitoring in VBT protocols (Weakley et al., 2021). Two sets were performed with a 1-min rest interval between sets. This interval kept the two-set conditioning sequence brief and was applied consistently across all conditions. No predetermined number of repetitions was prescribed. Instead, each set was terminated when the velocity loss of a repetition first reached or exceeded the prescribed threshold relative to the fastest repetition within that set. Specifically, the set was terminated at velocity-loss thresholds of 10%, 20%, and 30% in the VL10%, VL20%, and VL30% conditions, respectively (Rodiles-Guerrero et al., 2020; Hernández-Belmonte and Pallarés, 2022; Li et al., 2024).

Straight-punch performance was assessed at baseline (T0) and at 4, 8, 12, and 16 min following the conditioning protocol (T1–T4, respectively). At each assessment, participants performed three maximal-effort straight punches with the lead-hand and three with the rear hand from their habitual boxing stance. The assessment sequence commenced at the prespecified time point, and the order of lead- and rear-hand testing was kept consistent across all experimental sessions for each participant. A fixed 15-s recovery interval was provided between successive punch attempts to minimize the influence of repeated-effort fatigue. The striking target was positioned at shoulder height, and the starting distance was individualized according to each athlete’s functional arm span and marked on the floor to ensure consistent positioning across sessions. A punch attempt was considered valid only when the prescribed straight punch was delivered from the marked starting position, contacted the target, and yielded usable force and velocity recordings from the same attempt; attempts that did not meet these criteria were excluded from analysis. For each hand, the highest valid value obtained from the three attempts was retained for subsequent analysis.

To minimize the influence of equipment variability on mechanical output, boxing gloves were standardized according to the International Boxing Association (IBA) “Technical & Competition Rules.” Male boxers used 10-ounce gloves from the Minimum Weight (46–48 kg) category to the Welterweight (67 kg) category, whereas 12-ounce gloves were used from the Light Middleweight (71 kg) category to the Super Heavyweight (>92 kg) category. Throughout the study, glove specifications were assigned according to each participant’s weight category and kept consistent across all experimental sessions.

### 2.4 Measurement indicators

The primary performance outcomes were peak punch force (PPF), and peak punch velocity (PPV). An estimated peak punch-power index (PPP) was calculated as a secondary composite outcome.

#### 2.4.1 PPF (N)

Force signals during the straight punching test were collected using an Innolife boxing force-measuring target (InnoLife Technology, USA). The force-measuring target was positioned at shoulder height and oriented vertically to the ground, with a sampling frequency of 1000 Hz. PPF (in N) was defined as the instantaneous maximum value of the force-time curve.

#### 2.4.2 PPV (m/s)

Velocity signals were obtained using a Hykso wearable boxing sensor (Hykso Inc., USA). The sensor has previously demonstrated acceptable validity and reliability for estimating punch velocity-related metrics and punch counts in boxing research (Omcirk et al., 2021; Omcirk et al., 2023). The sensor was secured to the distal forearm, proximal to the wrist joint, using an adjustable strap to ensure stable positioning and measurement consistency across trials. The device generated a velocity–time signal, from which PPV was defined as the maximal instantaneous velocity recorded during the punch.

#### 2.4.3 PPP (W)

For each valid punch attempt, peak punch force (PPF; N) obtained from the force-measuring target and peak punch velocity (PPV; m/s) obtained from the wearable sensor were paired within the same punch attempt. The estimated peak punch-power index (PPP; W) was calculated separately for each attempt as follows:

PPP (W) = PPF (N) × PPV (m/s).

Although PPF and PPV were obtained from the same punch attempt, force and velocity were recorded independently using separate devices and were not synchronized at the waveform level. Because peak force and peak velocity may have occurred at different instants during the punching action, PPP does not represent directly measured instantaneous mechanical power. Accordingly, PPP was interpreted as an estimated same-attempt force–velocity product, or punch-power index. The highest valid PPP value among the three punch attempts was retained for statistical analysis.

### 2.5 Statistical analyses

All statistical analyses were performed using R version 4.3.1. Data are presented as mean ± standard deviation (SD). Outcomes were analyzed using separate linear mixed-effects models (lme4 and lmerTest packages), with condition (CON, VL10%, VL20%, and VL30%), time (T0–T4), and the condition × time interaction included as fixed effects, and participant ID included as a random intercept. Models were estimated using restricted maximum likelihood. Sum contrasts and Type III F tests were used, with denominator degrees of freedom estimated using the Kenward–Roger approximation.

For outcomes showing a significant condition × time interaction, time-specific comparisons were conducted by fitting a separate mixed-effects model at each assessment time point, with condition as a fixed effect and participant as a random intercept. Estimated marginal means were compared using Dunnett-adjusted contrasts between each VL condition and CON. These contrasts evaluated time-specific differences from CON; separate change-from-baseline contrasts were not performed. For outcomes without a significant condition × time interaction, time-specific VL-versus-CON contrasts were additionally estimated for descriptive completeness but were treated as exploratory and were not used to support inferential conclusions. Standardized effect sizes were calculated as Hedges’ g by dividing each estimated VL-versus-CON contrast by the residual SD of the corresponding time-specific model and applying the small-sample correction J = 1-3/(4df-1). The confidence limits for Hedges’ g were obtained by applying the same standardization and correction to the confidence limits of the corresponding estimated mean difference. Effect sizes were interpreted as small (0.2), moderate (0.5), and large (0.8). All statistical tests were two-sided, with statistical significance set at *p* < 0.05. Using the same time-specific linear mixed-effects models as the Dunnett comparisons, additional *post hoc* pairwise comparisons among VL10%, VL20%, and VL30% were conducted at each time point. Tukey adjustment was applied to the three pairwise comparisons within each time point, and estimated differences, 95% confidence intervals, and *p* values were reported. These analyses were considered exploratory.

## 3 Results

### 3.1 Training volume characteristics

The number of repetitions completed increased progressively as a greater velocity-loss threshold was permitted. In the VL10% condition, participants completed 2.4 ± 0.5 repetitions in Set 1 and 2.2 ± 0.4 repetitions in Set 2, corresponding to 4.6 ± 0.8 repetitions across the two sets. The corresponding values were 3.1 ± 0.7 and 2.8 ± 0.5 repetitions for VL20% (5.9 ± 1.1 repetitions in total), and 3.7 ± 0.8 and 3.2 ± 0.7 repetitions for VL30% (6.9 ± 1.4 repetitions in total). Volume-load increased accordingly across the VL conditions, from 339.4 ± 81.5 kg in VL10% to 432.6 ± 104.7 kg in VL20% and 508.8 ± 142.9 kg in VL30%. Descriptively, repetition counts were slightly lower in the second set than in the first set across all VL conditions. CON comprised a fixed total of 20 repetitions (two sets of 10); however, its mean external load and volume-load could not be reported because exact participant-level loads were not retained. Set-specific repetition counts, total repetitions, and volume-load for each VL condition are presented in **Supplementary Table S1**.

### 3.2 Peak punching velocity

For lead-hand PPV, the condition × time interaction was not significant, F(12, 323) = 1.06, *p* = 0.391; therefore, the time-specific VL-versus-CON contrasts were treated as exploratory. In contrast, rear-hand PPV showed a significant condition × time interaction, F(12, 323) = 5.21, *p* < 0.001. Relative to CON, VL30% showed lower rear-hand PPV at 4 and 8 min (*p* ≤ 0.011; Hedges’ g = −2.118 to −1.000), whereas VL10% and VL20% showed higher values at 8 and 12 min (*p* ≤ 0.034; g = 0.855–0.963). No significant VL-versus-CON differences were observed at baseline or 16 min (*p* > 0.071). The corresponding PPV distributions across conditions and assessment times are presented in **Figure 2**.

**Figure 2:**
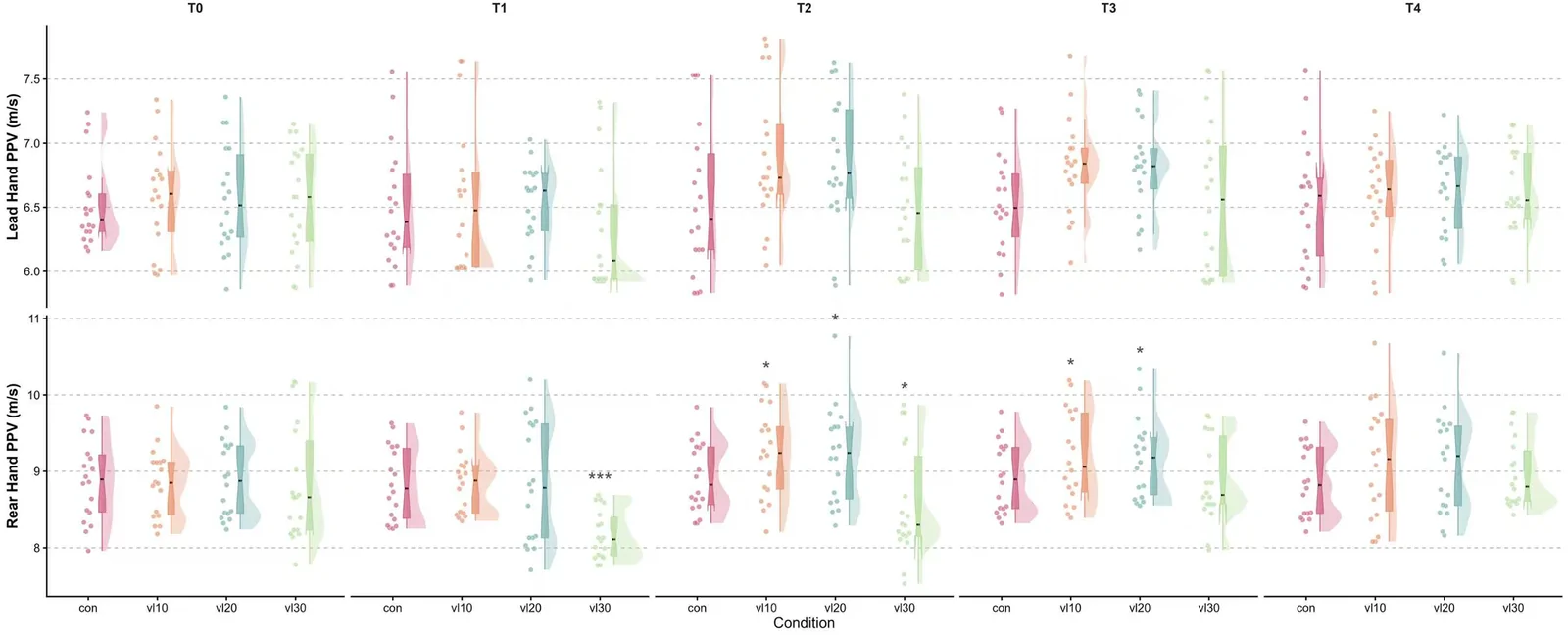
Raincloud plots of lead-hand (upper panel) and rear-hand (lower panel) peak punch velocity across conditions and time points. * and *** indicate Dunnett-adjusted differences from CON at p < 0.05 and p < 0.001, respectively. Because the condition × time interaction was not significant for lead-hand PPV, the contrasts in the upper panel are exploratory. T0–T4: baseline and 4, 8, 12, and 16 min post-conditioning, respectively.

### 3.3 Peak punching force

Significant condition × time interactions were observed for both lead-hand PPF, F(12, 323) = 2.79, *p* = 0.001, and rear-hand PPF, F(12, 323) = 3.17, *p* < 0.001. For the lead hand, VL30% showed lower PPF than CON at 4, 8, and 12 min (*p* ≤ 0.046; Hedges’ g = −1.880 to −0.814), whereas VL10% and VL20% did not differ significantly from CON. For the rear hand, VL30% showed lower PPF at 4 min (*p* < 0.001; g = −1.512), while VL10% and VL20% showed higher values at 8 and 12 min (*p* ≤ 0.039; g = 0.835–1.001). No significant VL-versus-CON differences were observed at baseline or 16 min (*p* > 0.233). The corresponding PPF distributions across conditions and assessment times are presented in **Figure 3**.

**Figure 3:**
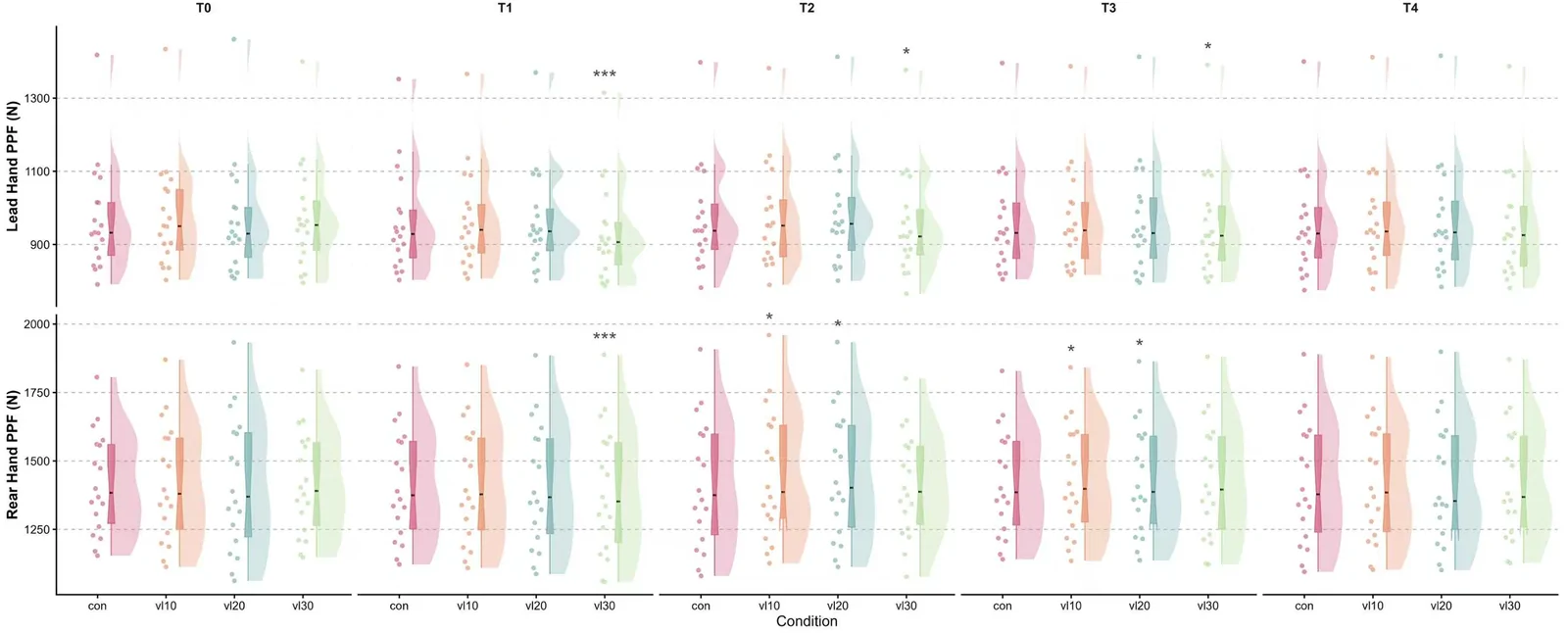
Raincloud plots of lead-hand (upper panel) and rear-hand (lower panel) peak punch force across conditions and time points. * and *** indicate Dunnett-adjusted differences from CON at p < 0.05 and p < 0.001, respectively. T0–T4: baseline and 4, 8, 12, and 16 min post-conditioning, respectively.

### 3.4 Peak punch-power index

Significant condition × time interactions were observed for both lead-hand PPP, F(12, 323) = 1.81, *p* = 0.046, and rear-hand PPP, F(12, 323) = 7.39, *p* < 0.001. Lead-hand PPP was higher under VL10% and VL20% than under CON at 8 and 12 min (*p* ≤ 0.024; Hedges’ g = 0.902–1.000), with no significant differences at the other assessment times. For the rear hand, VL30% showed lower PPP than CON at 4 and 8 min (*p* ≤ 0.032; g = −2.439 to −0.861), whereas VL10% and VL20% showed higher values at 8 and 12 min (*p* ≤ 0.011; g = 0.999–1.267). No significant VL-versus-CON differences were observed at baseline or 16 min (*p* ≥ 0.062). The corresponding PPP distributions across conditions and assessment times are presented in **Figure 4**.

**Figure 4:**
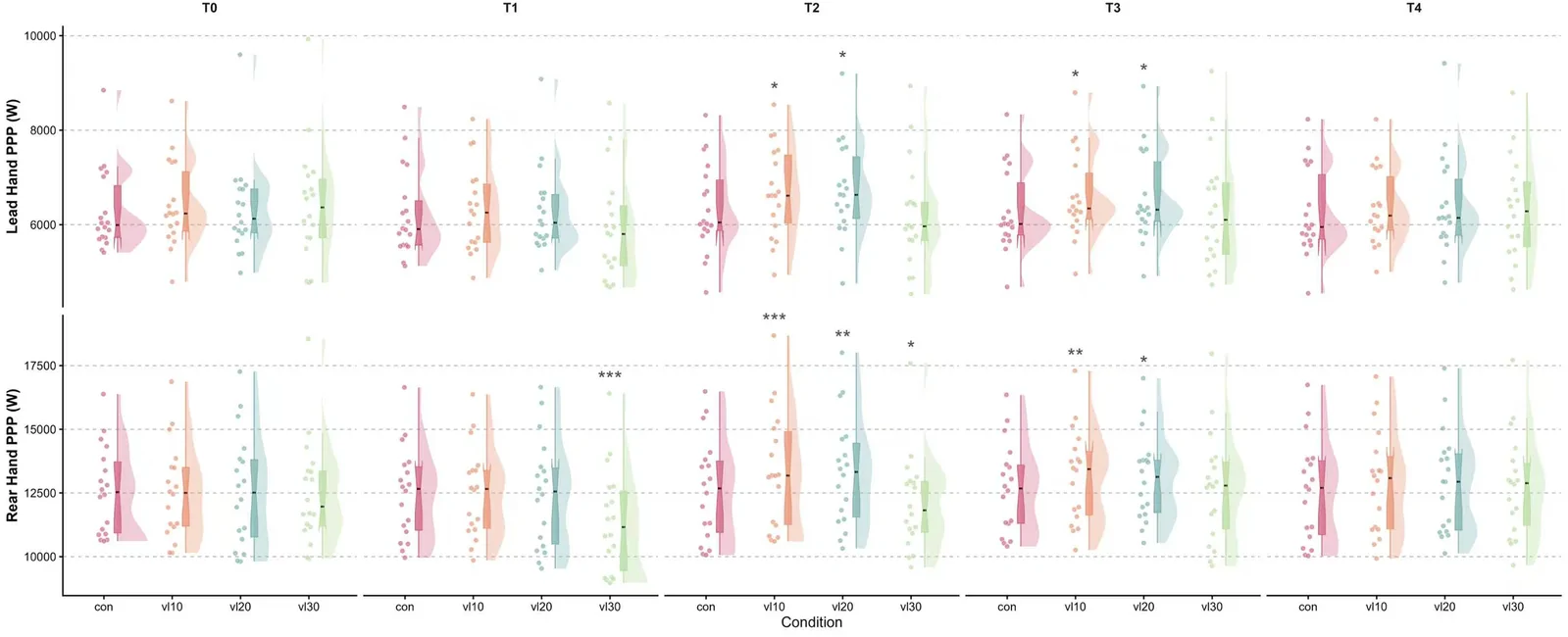
Raincloud plots of lead-hand (upper panel) and rear-hand (lower panel) peak punch-power index across conditions and time points. *, **, and *** indicate Dunnett-adjusted differences from CON at *p* < 0.05, *p* < 0.01, and *p* < 0.001, respectively. T0–T4: baseline and 4, 8, 12, and 16 min post-conditioning, respectively.

Complete descriptive statistics, model outputs, and Dunnett-adjusted VL-versus-CON contrast estimates are provided in **Supplementary Tables S2**-**S4**.

### 3.5 Pairwise comparisons among VL conditions

No significant differences were observed at T0 or between VL10% and VL20% at any time point for either hand or any outcome. All significant differences involved VL30%, with higher values under VL10% or VL20%.

For the lead hand, PPF was significantly higher under both VL10% and VL20% than under VL30% at T1–T3 (estimated differences: 12.06–34.22 N; *p* ≤ 0.003). At T4, PPF was also higher under VL10% than under VL30% (estimated difference: 13.28 N; *p* = 0.026). PPV was significantly higher under both VL10% and VL20% than under VL30% at T2 (estimated differences: 0.37–0.39 m·s^-1^; *p* = 0.008 and 0.013, respectively). PPP was significantly higher under both VL10% and VL20% than under VL30% at T1–T3 (estimated differences: 275.89–516.61 W; *p* ≤ 0.045).

For the rear hand, PPF was significantly higher under both VL10% and VL20% than under VL30% at T1 and T2 (estimated differences: 21.94–37.50 N; *p* ≤ 0.013). PPV was significantly higher under both VL10% and VL20% than under VL30% at T1–T3 (estimated differences: 0.30–0.69 m·s^-1^; *p* ≤ 0.006). Similarly, PPP was significantly higher under both VL10% and VL20% than under VL30% at T1–T3 (estimated differences: 410.83–1281.56 W; *p* ≤ 0.014). All other pairwise comparisons among the VL conditions were not significant. Complete estimated differences, 95% confidence intervals, and *p* values are presented in **Supplementary Tables S5**, **S6**.

## 4 Discussion

The present study investigated the acute effects of bench press conditioning performed with different velocity loss thresholds on straight-punch performance in young boxers. The main findings were as follows. First, VL10% and VL20% showed favorable time-specific differences relative to CON for several punch-performance outcomes, particularly at 8 and 12 min post-conditioning. Specifically, both conditions showed higher rear-hand PPV, PPF, and PPP at 8 and 12 min, as well as higher lead-hand PPP at these time points. In contrast, neither VL10% nor VL20% showed significant positive differences from CON for lead-hand PPF, while lead-hand PPV did not show a significant condition × time interaction and its time-specific contrasts were therefore treated as exploratory. Direct pairwise comparisons showed no significant differences between VL10% and VL20% at any time point, whereas both conditions produced higher values than VL30% for several outcomes at selected time points. Conversely, VL30% showed lower values than CON for several outcomes during the early post-conditioning period, particularly at 4 min and, for some outcomes, at 8–12 min. Second, the observed response pattern was time dependent, with favorable differences relative to CON occurring most consistently at 8 and 12 min, whereas most differences were no longer evident by 16 min. Third, the favorable time-specific differences were descriptively more apparent across rear-hand outcomes. However, because response magnitude was not directly compared statistically between the lead- and rear-hands, this pattern should not be interpreted as evidence of a significantly greater PAPE response in the rear-hand. Taken together, these findings suggest that VL-regulated bench press conditioning was associated with distinct time-dependent punch-performance responses, although the independent influence of VL threshold cannot be separated from differences in repetition volume and conditioning dose.

The favorable time-specific differences relative to CON observed under the VL10% and VL20% conditions may reflect a more favorable balance between potentiation and fatigue; however, this interpretation remains speculative because fatigue-related responses were not directly assessed and the conditioning dose differed among the VL conditions. When the conditioning stimulus induces sufficient neuromuscular activation without excessive fatigue, potentiation is more likely to be expressed in subsequent explosive actions (Tillin and Bishop, 2009). In the present study, the lower-volume VL10% and VL20% protocols may have provided sufficient neuromuscular stimulation while limiting fatigue exposure relative to VL30%, thereby contributing to a more favorable stimulus–recovery balance. Conversely, the greater repetition volume and volume-load associated with higher VL thresholds, particularly VL30%, may have contributed to greater residual fatigue and to the lower values observed relative to CON during the earlier post-conditioning period. Previous studies have also shown that, under the same external load, larger velocity loss values are typically associated with higher blood lactate, greater perceived exertion, and larger reductions in power output (Weakley et al., 2020). In addition, Sánchez-Medina and González-Badillo reported that greater velocity loss significantly decreases movement velocity (Sánchez-Medina and González-Badillo, 2011), and Pareja-Blanco et al. demonstrated that higher velocity loss thresholds can induce greater neuromuscular fatigue and negatively affect explosive performance (Pareja-Blanco et al., 2017). The present findings are generally consistent with previous studies and suggest that lower-dose VL-regulated bench press conditioning may be associated with a more favorable stimulus–recovery balance for subsequent explosive performance. However, because repetition volume and volume-load increased with the prescribed VL threshold, the independent contribution of VL threshold cannot be separated from the accompanying differences in conditioning dose. A further consideration is that CON consisted of 20 repetitions performed using a habitual warm-up load below 30% 1RM. Although the relative load was low, this protocol may have induced some fatigue. Because exact participant-level loads used during CON were not retained, its volume-load could not be calculated or directly compared with those of the VL conditions. Therefore, the observed VL-versus-CON differences should be interpreted as comparisons between active bench press protocols rather than as effects relative to a passive or demonstrably non-fatiguing control condition.

In the present study, favorable VL-versus-CON differences were most consistently observed at 8 and 12 min post-conditioning across several punch-performance outcomes. This temporal pattern may reflect the time-dependent interaction between the dissipation of fatigue and the persistence of potentiation, although these mechanisms were not directly assessed. The time course of recovery following resistance exercise is influenced by the magnitude of velocity loss in the set, with greater VL requiring longer recovery periods before performance returns to baseline (Pareja-Blanco et al., 2019). Previous studies have indicated that the optimal recovery time for PAPE is influenced by several factors, including training status and stimulus intensity. A meta-analysis by Wilson et al. reported that following high-intensity conditioning stimuli, a recovery period of approximately 7–10 min typically produces favorable enhancement effects (Wilson et al., 2013). Furthermore, Seitz & Haff suggested that for highly trained athletes, slightly longer recovery intervals may allow greater fatigue reduction, thereby potentially allowing potentiation effects to be more fully expressed (Seitz and Haff, 2016). The findings of the present study are generally consistent with this previous observation.

In addition, favorable time-specific differences relative to CON were descriptively more apparent across rear-hand outcomes. For rear-hand PPV, a significant condition × time interaction was observed, with VL10% and VL20% showing significantly higher values than CON at 8 and 12 min post-conditioning. In contrast, lead-hand PPV did not exhibit a significant condition × time interaction. Although exploratory Dunnett-adjusted VL-versus-CON contrasts were reported for descriptive completeness, these comparisons were not interpreted inferentially; therefore, a clear condition-specific PAPE response could not be confirmed for lead-hand PPV. This descriptive difference in response patterns between hands may be related to the biomechanical characteristics of boxing movements. Previous research has shown that the rear-hand straight punch typically involves a longer acceleration path and can generate greater peak force and velocity outputs (Lenetsky et al., 2013). Therefore, when conditioning activities enhance neuromuscular activation, the potentiation effect may be more readily expressed during the rear-hand straight punch, which typically involves a longer acceleration pathway and greater force production than the lead-hand punch (Turner et al., 2011). Compared with the rear-hand straight punch, the lead-hand straight punch generally involves a shorter movement trajectory and lower velocity outputs (Piorkowski et al., 2011; Wan and Liu, 2026), which may partly explain the less consistent favorable time-specific differences observed for lead-hand outcomes in the present study.

It is noteworthy that athletes from different sports and movement tasks may respond differently to velocity loss (VL) thresholds. Previous studies have reported different optimal VL thresholds and recovery windows depending on the testing task and athlete population. For example, Cui et al. reported that in boxers, the dominant and non-dominant sides showed different optimal VL thresholds and recovery intervals for punching performance (Cui et al., 2024). In contrast, Yuan et al. observed in track and field athletes that only a very low VL threshold (5%) significantly enhanced countermovement jump performance after conditioning (Yuan et al., 2022). In the present study, favorable time-specific differences relative to CON were observed under both VL10% and VL20%, whereas VL30% showed lower values than CON for several outcomes during the earlier recovery period. Taken together, findings from previous studies and the present results suggest that performance responses to VL-regulated conditioning may vary according to task type, movement characteristics, conditioning dose, and individual athlete characteristics.

The present findings provide practical information for the use of VL-regulated bench press conditioning in boxing. Under the present protocol, VL10% and VL20% showed favorable time-specific differences relative to CON for several punch-performance outcomes, particularly at 8 and 12 min after the conditioning activity, whereas VL30% showed lower values than CON for several outcomes during the earlier recovery period. For similarly trained male adolescent boxers, coaches may consider allowing approximately 8–12 min of recovery between high-load bench press conditioning at 85% 1RM and subsequent explosive punching tasks. Although both VL10% and VL20% produced higher values than VL30% for several outcomes at selected time points, VL10% and VL20% did not differ significantly from each other. Because the conditions differed in total repetition volume, these findings do not isolate the effect of VL threshold or establish an optimal threshold. In addition, the practical relevance of smaller between-condition differences should be interpreted cautiously because study-specific measurement-error thresholds were not established. Therefore, VL-regulated conditioning should be individualized according to the athlete’s response and implemented with consideration of competition schedules, equipment availability, and practical warm-up constraints.

The present study had some limitations that should be acknowledged. First, the participants were male boxers aged 16–19 years, including seven younger than 18 years. Biological maturation was not assessed, so we could not determine whether maturity status contributed to variation in punch performance or acute responses to bench press conditioning. The findings should therefore be generalized cautiously to adolescent boxers at other stages of maturation, as well as to female and older adult boxers. Second, the modest sample size may have reduced the precision of the estimated effects. Third, CON was an active bench press condition rather than a passive control, and exact participant-level CON loads were not retained, preventing direct comparison of its volume-load with those of the VL conditions. In addition, the VL conditions were not volume-matched. Because higher VL thresholds resulted in greater total repetition volume, the independent effect of VL threshold cannot be separated from that of conditioning dose and associated fatigue exposure. The fixed 1-min inter-set rest may have limited recovery before the second set, particularly in the VL30% condition; therefore, the observed punch responses should be interpreted in the context of this rest interval. Fourth, no direct neuromuscular, metabolic, perceptual, or autonomic measures of fatigue or potentiation were collected; therefore, the proposed potentiation–fatigue explanations remain inferential. In addition, bench press was used as an upper-body conditioning activity, whereas punching depends on coordinated lower-limb–trunk–upper-limb force transfer. Fifth, PPP was derived from same-attempt peak force and peak velocity recorded using separate devices without waveform-level synchronization and should therefore be interpreted as an estimated force–velocity product rather than direct instantaneous mechanical power. Published validation data for the specific punch-force target were unavailable, so the accuracy of the absolute PPF values remains uncertain. Formal test–retest reliability and measurement-error indices were also unavailable, so it remains uncertain whether all smaller statistically significant differences exceeded normal measurement variability. Finally, the study examined only acute responses, and the findings should not be extrapolated to chronic adaptations.

## 5 Conclusion

The present study showed that VL-regulated bench press conditioning was associated with time-dependent differences in straight-punch performance in the trained male adolescent boxers studied. Relative to the active control condition, VL10% and VL20% showed favorable differences for several punch-performance outcomes at 8 and 12 min post-conditioning, whereas VL30% showed lower values than CON for several outcomes during the earlier recovery period. These time-specific differences were generally more evident for the rear-hand straight punch. Direct pairwise comparisons revealed no significant differences between VL10% and VL20%, whereas both conditions produced higher values than VL30% for several outcomes at selected time points. Furthermore, because the VL conditions were not volume-matched, the observed responses should be interpreted as reflecting the combined influence of VL threshold, repetition volume, conditioning dose, and associated fatigue exposure rather than the isolated effect of VL threshold. Collectively, these findings suggest that VL-regulated bench press conditioning may inform warm-up design for similarly trained male adolescent boxers, with the 8–12 min post-conditioning period representing a practically relevant recovery window under the present experimental protocol. Whether the same responses occur at other stages of biological maturation remains unknown.

## Data Availability

The original contributions presented in the study are included in the article/**Supplementary Material**. Further inquiries can be directed to the corresponding authors.
