# Supplementary material for: Acute effects of bench press conditioning performed with different velocity-loss thresholds on punch performance in elite adolescent boxers
Source: Front Physiol. 2026 Oct 14;17:1973551. doi: 10.3389/fphys.2026.1973551 (PMC13713327; doi:10.3389/fphys.2026.1973551)
Supplement: Supplementary file 1 [file Table1.docx]

Supplementary Material

Supplementary Table S1. Set-specific repetitions, total repetitions, and volume-load across the control and VL conditions.

| Condition | Relative load | Set 1 | Set 2 | Total across two sets | Volume-load (kg) |
| --- | --- | --- | --- | --- | --- |
| CON | <30% 1RM | 10 | 10 | 20 | Not reported |
| VL10% | 85% 1RM | 2.4 ± 0.5 | 2.2 ± 0.4 | 4.6 ± 0.8 | 339.4 ± 81.5 |
| VL20% | 85% 1RM | 3.1 ± 0.7 | 2.8 ± 0.5 | 5.9 ± 1.1 | 432.6 ± 104.7 |
| VL30% | 85% 1RM | 3.7 ± 0.8 | 3.2 ± 0.7 | 6.9 ± 1.4 | 508.8 ± 142.9 |

Note. Repetition counts for CON were fixed by the experimental protocol, whereas repetition counts for the VL conditions are presented as mean ± SD. In the VL conditions, each set was terminated when the velocity loss of a repetition first reached or exceeded the prescribed threshold relative to the fastest repetition within that set. The threshold-triggering repetition was included in the completed repetition count. Total repetitions were calculated as the sum of repetitions completed across the two sets. For the VL conditions, volume-load was calculated at the participant level as the individualized external load multiplied by the total number of repetitions completed across both sets and was subsequently summarized as mean ± SD. Loads corresponding to 85% 1RM were rounded to the nearest 2.5 kg. Volume-load for CON was not reported because exact participant-level external loads were not retained. CON, active control; VL, velocity loss; 1RM, one-repetition maximum.

Supplementary Table S2. Descriptive statistics (mean ± standard deviation) for each indicator across time points (T0–T4) and conditions (CON, VL10%, VL20%, VL30%).

| Indicator | Time | CON | VL10% | VL20% | VL30% |
| --- | --- | --- | --- | --- | --- |
| Lead Hand PPV | T0 | 6.52 ± 0.33 | 6.58 ± 0.42 | 6.57 ± 0.42 | 6.56 ± 0.42 |
|  | T1 | 6.50 ± 0.48 | 6.56 ± 0.57 | 6.53 ± 0.31 | 6.33 ± 0.50 |
|  | T2 | 6.55 ± 0.59 | 6.88 ± 0.54 | 6.86 ± 0.51 | 6.49 ± 0.47 |
|  | T3 | 6.54 ± 0.40 | 6.82 ± 0.38 | 6.81 ± 0.36 | 6.57 ± 0.59 |
|  | T4 | 6.54 ± 0.49 | 6.61 ± 0.38 | 6.63 ± 0.33 | 6.61 ± 0.36 |
| Rear Hand PPV | T0 | 8.89 ± 0.53 | 8.85 ± 0.45 | 8.90 ± 0.50 | 8.83 ± 0.77 |
|  | T1 | 8.84 ± 0.48 | 8.86 ± 0.42 | 8.84 ± 0.80 | 8.17 ± 0.31 |
|  | T2 | 8.92 ± 0.46 | 9.23 ± 0.59 | 9.22 ± 0.63 | 8.58 ± 0.73 |
|  | T3 | 8.92 ± 0.46 | 9.19 ± 0.60 | 9.17 ± 0.52 | 8.87 ± 0.55 |
|  | T4 | 8.86 ± 0.46 | 9.12 ± 0.76 | 9.11 ± 0.66 | 8.96 ± 0.45 |
| Lead Hand PPF | T0 | 967.94 ± 146.64 | 977.94 ± 147.28 | 965.56 ± 155.44 | 973.39 ± 145.11 |
|  | T1 | 959.89 ± 139.70 | 966.56 ± 137.66 | 963.44 ± 135.30 | 932.33 ± 133.52 |
|  | T2 | 971.22 ± 142.61 | 974.78 ± 143.71 | 982.61 ± 147.72 | 954.67 ± 142.26 |
|  | T3 | 963.39 ± 144.60 | 969.44 ± 141.35 | 968.06 ± 153.06 | 956.00 ± 145.06 |
|  | T4 | 958.56 ± 147.91 | 966.94 ± 147.93 | 965.39 ± 151.04 | 953.67 ± 148.21 |
| Rear Hand PPF | T0 | 1418.89 ± 185.04 | 1421.28 ± 214.88 | 1416.56 ± 247.06 | 1417.89 ± 192.28 |
|  | T1 | 1414.28 ± 204.96 | 1417.11 ± 215.70 | 1413.67 ± 227.04 | 1391.72 ± 235.05 |
|  | T2 | 1417.78 ± 235.03 | 1450.33 ± 235.34 | 1448.06 ± 234.97 | 1412.83 ± 190.11 |
|  | T3 | 1422.39 ± 195.08 | 1433.56 ± 201.03 | 1432.11 ± 202.19 | 1428.78 ± 215.02 |
|  | T4 | 1420.78 ± 225.08 | 1422.17 ± 220.48 | 1422.61 ± 225.85 | 1425.28 ± 211.99 |
| Lead Hand PPP | T0 | 6293.44 ± 871.26 | 6423.33 ± 917.20 | 6328.06 ± 1001.86 | 6417.39 ± 1253.16 |
|  | T1 | 6226.89 ± 933.90 | 6326.33 ± 916.51 | 6287.17 ± 928.30 | 5929.28 ± 1138.04 |
|  | T2 | 6344.39 ± 953.02 | 6693.39 ± 962.38 | 6730.56 ± 1020.50 | 6213.94 ± 1133.03 |
|  | T3 | 6283.50 ± 887.50 | 6604.22 ± 923.38 | 6580.83 ± 971.78 | 6304.94 ± 1227.65 |
|  | T4 | 6250.06 ± 941.30 | 6363.17 ± 815.38 | 6402.22 ± 1065.65 | 6316.61 ± 1090.12 |
| Rear Hand PPP | T0 | 12596.83 ± 1725.31 | 12557.67 ± 1866.91 | 12584.11 ± 2223.93 | 12514.44 ± 2080.11 |
|  | T1 | 12481.17 ± 1804.24 | 12519.44 ± 1780.85 | 12475.83 ± 2140.83 | 11372.61 ± 2052.31 |
|  | T2 | 12622.94 ± 2011.76 | 13386.00 ± 2312.44 | 13314.39 ± 2143.67 | 12104.44 ± 1889.70 |
|  | T3 | 12672.67 ± 1771.57 | 13148.72 ± 1841.72 | 13099.89 ± 1718.67 | 12689.06 ± 2155.98 |
|  | T4 | 12578.44 ± 2014.01 | 12929.44 ± 2046.84 | 12916.56 ± 2028.45 | 12772.39 ± 2032.65 |

Supplementary Table S3. Type III mixed-effects analysis of variance (ANOVA) results for the effects of condition, time, and their interaction on each punching performance indicator.

| indicator | Effect | NumDF | DenDF | F value | *p* value |
| --- | --- | --- | --- | --- | --- |
| Lead Hand PPV | Condition | 3 | 323 | 6.02 | <0.001 |
|  | Time | 4 | 323 | 4.30 | 0.002 |
|  | Condition×Time | 12 | 323 | 1.06 | 0.391 |
| Rear Hand PPV | Condition | 3 | 323 | 30.98 | <0.001 |
|  | Time | 4 | 323 | 18.22 | <0.001 |
|  | Condition×Time | 12 | 323 | 5.21 | <0.001 |
| Lead Hand PPF | Condition | 3 | 323 | 16.16 | <0.001 |
|  | Time | 4 | 323 | 9.80 | <0.001 |
|  | Condition×Time | 12 | 323 | 2.79 | 0.001 |
| Rear Hand PPF | Condition | 3 | 323 | 7.37 | <0.001 |
|  | Time | 4 | 323 | 11.94 | <0.001 |
|  | Condition×Time | 12 | 323 | 3.17 | <0.001 |
| Lead Hand PPP | Condition | 3 | 323 | 10.11 | <0.001 |
|  | Time | 4 | 323 | 6.87 | <0.001 |
|  | Condition×Time | 12 | 323 | 1.81 | 0.046 |
| Rear Hand PPP | Condition | 3 | 323 | 34.22 | <0.001 |
|  | Time | 4 | 323 | 26.78 | <0.001 |
|  | Condition×Time | 12 | 323 | 7.39 | <0.001 |

Supplementary Table S4. Post hoc pairwise comparisons (Dunnett's test) of each velocity-loss condition (VL10%, VL20%, VL30%) against the control condition (CON) at each time point (T0–T4), with effect sizes (Hedges' g) and 95% confidence intervals.

| Indicator | Time | Condition | Difference [95% CI] | Hedges g [95% CI] | SE | df | t ratio | *p* value |
| --- | --- | --- | --- | --- | --- | --- | --- | --- |
| Lead Hand PPV | T0 | VL10% | 0.063 [-0.250, 0.375] | 0.161 [-0.641, 0.963] | 0.128 | 66.000 | 0.489 | 0.901 |
|  | T0 | VL20% | 0.049 [-0.263, 0.361] | 0.126 [-0.676, 0.927] | 0.128 | 66.000 | 0.381 | 0.940 |
|  | T0 | VL30% | 0.039 [-0.273, 0.351] | 0.100 [-0.702, 0.902] | 0.128 | 66.000 | 0.303 | 0.963 |
|  | T1 | VL10% | 0.063 [-0.303, 0.429] | 0.139 [-0.663, 0.941] | 0.150 | 66.000 | 0.421 | 0.927 |
|  | T1 | VL20% | 0.029 [-0.337, 0.395] | 0.063 [-0.739, 0.865] | 0.150 | 66.000 | 0.192 | 0.985 |
|  | T1 | VL30% | -0.168 [-0.534, 0.198] | -0.367 [-1.169, 0.434] | 0.150 | 66.000 | -1.115 | 0.543 |
|  | T2 | VL10% | 0.336 [0.029, 0.642] | 0.878 [0.076, 1.680] | 0.126 | 66.000 | 2.665 | 0.028 |
|  | T2 | VL20% | 0.313 [0.007, 0.620] | 0.820 [0.018, 1.622] | 0.126 | 66.000 | 2.489 | 0.044 |
|  | T2 | VL30% | -0.058 [-0.364, 0.249] | -0.151 [-0.953, 0.651] | 0.126 | 66.000 | -0.459 | 0.913 |
|  | T3 | VL10% | 0.287 [0.015, 0.559] | 0.845 [0.043, 1.647] | 0.112 | 66.000 | 2.563 | 0.037 |
|  | T3 | VL20% | 0.276 [0.004, 0.548] | 0.814 [0.012, 1.616] | 0.112 | 66.000 | 2.469 | 0.046 |
|  | T3 | VL30% | 0.037 [-0.235, 0.309] | 0.108 [-0.694, 0.910] | 0.112 | 66.000 | 0.328 | 0.956 |
|  | T4 | VL10% | 0.072 [-0.159, 0.303] | 0.249 [-0.553, 1.051] | 0.095 | 66.000 | 0.755 | 0.767 |
|  | T4 | VL20% | 0.093 [-0.138, 0.324] | 0.324 [-0.478, 1.126] | 0.095 | 66.000 | 0.983 | 0.627 |
|  | T4 | VL30% | 0.074 [-0.157, 0.306] | 0.258 [-0.544, 1.060] | 0.095 | 66.000 | 0.784 | 0.750 |
| Lead Hand PPF | T0 | VL10% | 10.000 [-5.346, 25.346] | 0.523 [-0.279, 1.325] | 6.306 | 66.000 | 1.586 | 0.279 |
|  | T0 | VL20% | -2.389 [-17.735, 12.958] | -0.125 [-0.927, 0.677] | 6.306 | 66.000 | -0.379 | 0.941 |
|  | T0 | VL30% | 5.444 [-9.902, 20.791] | 0.285 [-0.517, 1.086] | 6.306 | 66.000 | 0.863 | 0.702 |
|  | T1 | VL10% | 6.667 [-5.085, 18.419] | 0.455 [-0.347, 1.257] | 4.829 | 66.000 | 1.381 | 0.384 |
|  | T1 | VL20% | 3.556 [-8.196, 15.308] | 0.243 [-0.559, 1.045] | 4.829 | 66.000 | 0.736 | 0.778 |
|  | T1 | VL30% | -27.556 [-39.308, -15.804] | -1.880 [-2.682, -1.078] | 4.829 | 66.000 | -5.706 | <0.001 |
|  | T2 | VL10% | 3.556 [-10.350, 17.461] | 0.205 [-0.597, 1.007] | 5.714 | 66.000 | 0.622 | 0.839 |
|  | T2 | VL20% | 11.389 [-2.517, 25.295] | 0.657 [-0.145, 1.459] | 5.714 | 66.000 | 1.993 | 0.132 |
|  | T2 | VL30% | -16.556 [-30.461, -2.650] | -0.955 [-1.757, -0.153] | 5.714 | 66.000 | -2.897 | 0.016 |
|  | T3 | VL10% | 6.056 [-1.225, 13.336] | 0.667 [-0.135, 1.469] | 2.992 | 66.000 | 2.024 | 0.124 |
|  | T3 | VL20% | 4.667 [-2.614, 11.947] | 0.514 [-0.288, 1.316] | 2.992 | 66.000 | 1.560 | 0.291 |
|  | T3 | VL30% | -7.389 [-14.669, -0.109] | -0.814 [-1.616, -0.012] | 2.992 | 66.000 | -2.470 | 0.046 |
|  | T4 | VL10% | 8.389 [-3.677, 20.454] | 0.558 [-0.244, 1.360] | 4.958 | 66.000 | 1.692 | 0.233 |
|  | T4 | VL20% | 6.833 [-5.232, 18.899] | 0.454 [-0.348, 1.256] | 4.958 | 66.000 | 1.378 | 0.385 |
|  | T4 | VL30% | -4.889 [-16.954, 7.177] | -0.325 [-1.127, 0.477] | 4.958 | 66.000 | -0.986 | 0.625 |
| Lead Hand PPP | T0 | VL10% | 129.889 [-189.854, 449.632] | 0.326 [-0.476, 1.128] | 131.387 | 66.000 | 0.989 | 0.624 |
|  | T0 | VL20% | 34.611 [-285.132, 354.354] | 0.087 [-0.715, 0.889] | 131.387 | 66.000 | 0.263 | 0.972 |
|  | T0 | VL30% | 123.944 [-195.799, 443.688] | 0.311 [-0.491, 1.113] | 131.387 | 66.000 | 0.943 | 0.652 |
|  | T1 | VL10% | 99.444 [-254.919, 453.808] | 0.225 [-0.577, 1.027] | 145.613 | 66.000 | 0.683 | 0.807 |
|  | T1 | VL20% | 60.278 [-294.085, 414.641] | 0.136 [-0.666, 0.938] | 145.613 | 66.000 | 0.414 | 0.929 |
|  | T1 | VL30% | -297.611 [-651.974, 56.752] | -0.674 [-1.475, 0.128] | 145.613 | 66.000 | -2.044 | 0.119 |
|  | T2 | VL10% | 349.000 [39.344, 658.656] | 0.904 [0.102, 1.706] | 127.242 | 66.000 | 2.743 | 0.023 |
|  | T2 | VL20% | 386.167 [76.511, 695.822] | 1.000 [0.198, 1.802] | 127.242 | 66.000 | 3.035 | 0.011 |
|  | T2 | VL30% | -130.444 [-440.100, 179.211] | -0.338 [-1.140, 0.464] | 127.242 | 66.000 | -1.025 | 0.600 |
|  | T3 | VL10% | 320.722 [56.468, 584.976] | 0.973 [0.171, 1.775] | 108.586 | 66.000 | 2.954 | 0.013 |
|  | T3 | VL20% | 297.333 [33.079, 561.587] | 0.902 [0.100, 1.704] | 108.586 | 66.000 | 2.738 | 0.024 |
|  | T3 | VL30% | 21.444 [-242.810, 285.699] | 0.065 [-0.737, 0.867] | 108.586 | 66.000 | 0.197 | 0.985 |
|  | T4 | VL10% | 113.111 [-120.409, 346.631] | 0.388 [-0.414, 1.190] | 95.957 | 66.000 | 1.179 | 0.503 |
|  | T4 | VL20% | 152.167 [-81.354, 385.687] | 0.523 [-0.279, 1.325] | 95.957 | 66.000 | 1.586 | 0.279 |
|  | T4 | VL30% | 66.556 [-166.965, 300.076] | 0.229 [-0.573, 1.031] | 95.957 | 66.000 | 0.694 | 0.802 |
| Rear Hand PPV | T0 | VL10% | -0.036 [-0.296, 0.225] | -0.109 [-0.911, 0.693] | 0.107 | 66.000 | -0.332 | 0.955 |
|  | T0 | VL20% | 0.008 [-0.252, 0.269] | 0.026 [-0.776, 0.828] | 0.107 | 66.000 | 0.078 | 0.998 |
|  | T0 | VL30% | -0.060 [-0.321, 0.201] | -0.185 [-0.987, 0.617] | 0.107 | 66.000 | -0.560 | 0.869 |
|  | T1 | VL10% | 0.018 [-0.236, 0.273] | 0.058 [-0.744, 0.860] | 0.105 | 66.000 | 0.175 | 0.988 |
|  | T1 | VL20% | 0.006 [-0.249, 0.260] | 0.018 [-0.784, 0.819] | 0.105 | 66.000 | 0.053 | 0.999 |
|  | T1 | VL30% | -0.672 [-0.927, -0.418] | -2.118 [-2.920, -1.316] | 0.105 | 66.000 | -6.428 | <0.001 |
|  | T2 | VL10% | 0.309 [0.033, 0.586] | 0.897 [0.095, 1.699] | 0.114 | 66.000 | 2.721 | 0.025 |
|  | T2 | VL20% | 0.295 [0.018, 0.572] | 0.855 [0.053, 1.657] | 0.114 | 66.000 | 2.594 | 0.034 |
|  | T2 | VL30% | -0.345 [-0.622, -0.068] | -1.000 [-1.802, -0.198] | 0.114 | 66.000 | -3.034 | 0.011 |
|  | T3 | VL10% | 0.273 [0.046, 0.500] | 0.963 [0.161, 1.765] | 0.093 | 66.000 | 2.923 | 0.014 |
|  | T3 | VL20% | 0.256 [0.028, 0.483] | 0.903 [0.101, 1.704] | 0.093 | 66.000 | 2.739 | 0.024 |
|  | T3 | VL30% | -0.044 [-0.272, 0.183] | -0.157 [-0.959, 0.645] | 0.093 | 66.000 | -0.476 | 0.906 |
|  | T4 | VL10% | 0.254 [-0.017, 0.526] | 0.752 [-0.050, 1.554] | 0.111 | 66.000 | 2.283 | 0.071 |
|  | T4 | VL20% | 0.242 [-0.029, 0.513] | 0.716 [-0.086, 1.518] | 0.111 | 66.000 | 2.173 | 0.090 |
|  | T4 | VL30% | 0.097 [-0.174, 0.368] | 0.287 [-0.514, 1.089] | 0.111 | 66.000 | 0.872 | 0.697 |
| Rear Hand PPF | T0 | VL10% | 2.389 [-21.261, 26.039] | 0.081 [-0.721, 0.883] | 9.718 | 66.000 | 0.246 | 0.976 |
|  | T0 | VL20% | -2.333 [-25.984, 21.317] | -0.079 [-0.881, 0.723] | 9.718 | 66.000 | -0.240 | 0.977 |
|  | T0 | VL30% | -1.000 [-24.650, 22.650] | -0.034 [-0.836, 0.768] | 9.718 | 66.000 | -0.103 | 0.996 |
|  | T1 | VL10% | 2.833 [-9.134, 14.800] | 0.190 [-0.612, 0.992] | 4.917 | 66.000 | 0.576 | 0.862 |
|  | T1 | VL20% | -0.611 [-12.578, 11.356] | -0.041 [-0.843, 0.761] | 4.917 | 66.000 | -0.124 | 0.994 |
|  | T1 | VL30% | -22.556 [-34.523, -10.588] | -1.512 [-2.313, -0.710] | 4.917 | 66.000 | -4.587 | <0.001 |
|  | T2 | VL10% | 32.556 [3.478, 61.633] | 0.898 [0.096, 1.700] | 11.948 | 66.000 | 2.725 | 0.024 |
|  | T2 | VL20% | 30.278 [1.200, 59.355] | 0.835 [0.033, 1.637] | 11.948 | 66.000 | 2.534 | 0.039 |
|  | T2 | VL30% | -4.944 [-34.022, 24.133] | -0.136 [-0.938, 0.666] | 11.948 | 66.000 | -0.414 | 0.929 |
|  | T3 | VL10% | 11.167 [2.221, 20.112] | 1.001 [0.199, 1.803] | 3.676 | 66.000 | 3.038 | 0.011 |
|  | T3 | VL20% | 9.722 [0.777, 18.668] | 0.872 [0.070, 1.674] | 3.676 | 66.000 | 2.645 | 0.030 |
|  | T3 | VL30% | 6.389 [-2.557, 15.334] | 0.573 [-0.229, 1.375] | 3.676 | 66.000 | 1.738 | 0.215 |
|  | T4 | VL10% | 1.389 [-8.999, 11.777] | 0.107 [-0.695, 0.909] | 4.269 | 66.000 | 0.325 | 0.957 |
|  | T4 | VL20% | 1.833 [-8.555, 12.222] | 0.142 [-0.660, 0.943] | 4.269 | 66.000 | 0.429 | 0.924 |
|  | T4 | VL30% | 4.500 [-5.888, 14.888] | 0.347 [-0.455, 1.149] | 4.269 | 66.000 | 1.054 | 0.582 |
| Rear Hand PPP | T0 | VL10% | -39.167 [-488.920, 410.586] | -0.070 [-0.872, 0.732] | 184.810 | 66.000 | -0.212 | 0.982 |
|  | T0 | VL20% | -12.722 [-462.475, 437.031] | -0.023 [-0.825, 0.779] | 184.810 | 66.000 | -0.069 | 0.998 |
|  | T0 | VL30% | -82.389 [-532.142, 367.364] | -0.147 [-0.949, 0.655] | 184.810 | 66.000 | -0.446 | 0.918 |
|  | T1 | VL10% | 38.278 [-326.149, 402.704] | 0.084 [-0.718, 0.886] | 149.748 | 66.000 | 0.256 | 0.974 |
|  | T1 | VL20% | -5.333 [-369.760, 359.093] | -0.012 [-0.814, 0.790] | 149.748 | 66.000 | -0.036 | 1.000 |
|  | T1 | VL30% | -1108.556 [-1472.982, -744.129] | -2.439 [-3.241, -1.638] | 149.748 | 66.000 | -7.403 | <0.001 |
|  | T2 | VL10% | 763.056 [279.983, 1246.129] | 1.267 [0.465, 2.069] | 198.502 | 66.000 | 3.844 | 0.001 |
|  | T2 | VL20% | 691.444 [208.371, 1174.517] | 1.148 [0.346, 1.950] | 198.502 | 66.000 | 3.483 | 0.003 |
|  | T2 | VL30% | -518.500 [-1001.573, -35.427] | -0.861 [-1.663, -0.059] | 198.502 | 66.000 | -2.612 | 0.032 |
|  | T3 | VL10% | 476.056 [133.114, 818.997] | 1.113 [0.311, 1.915] | 140.920 | 66.000 | 3.378 | 0.004 |
|  | T3 | VL20% | 427.222 [84.281, 770.164] | 0.999 [0.197, 1.801] | 140.920 | 66.000 | 3.032 | 0.011 |
|  | T3 | VL30% | 16.389 [-326.553, 359.330] | 0.038 [-0.764, 0.840] | 140.920 | 66.000 | 0.116 | 0.995 |
|  | T4 | VL10% | 351.000 [-13.844, 715.844] | 0.772 [-0.030, 1.573] | 149.920 | 66.000 | 2.341 | 0.062 |
|  | T4 | VL20% | 338.111 [-26.733, 702.955] | 0.743 [-0.059, 1.545] | 149.920 | 66.000 | 2.255 | 0.075 |
|  | T4 | VL30% | 193.944 [-170.899, 558.788] | 0.426 [-0.376, 1.228] | 149.920 | 66.000 | 1.294 | 0.434 |

Supplementary Table S5. Tukey-adjusted pairwise comparisons among VL conditions for lead-hand outcomes.

| Outcome | Time | Comparison | Estimated difference | 95% CI | *p* value |
| --- | --- | --- | --- | --- | --- |
| PPF | T0 | VL10% - VL20% | 12.39 | [-2.83, 27.61] | 0.131 |
| PPF | T0 | VL10% - VL30% | 4.56 | [-10.67, 19.78] | 0.751 |
| PPF | T0 | VL20% - VL30% | -7.83 | [-23.06, 7.39] | 0.434 |
| PPF | T1 | VL10% - VL20% | 3.11 | [-8.55, 14.77] | 0.796 |
| PPF | T1 | VL10% - VL30% | 34.22 | [22.56, 45.88] | <0.001 |
| PPF | T1 | VL20% - VL30% | 31.11 | [19.45, 42.77] | <0.001 |
| PPF | T2 | VL10% - VL20% | -7.83 | [-21.63, 5.96] | 0.364 |
| PPF | T2 | VL10% - VL30% | 20.11 | [6.32, 33.90] | 0.003 |
| PPF | T2 | VL20% - VL30% | 27.94 | [14.15, 41.74] | <0.001 |
| PPF | T3 | VL10% - VL20% | 1.39 | [-5.83, 8.61] | 0.888 |
| PPF | T3 | VL10% - VL30% | 13.44 | [6.22, 20.67] | <0.001 |
| PPF | T3 | VL20% - VL30% | 12.06 | [4.83, 19.28] | <0.001 |
| PPF | T4 | VL10% - VL20% | 1.56 | [-10.41, 13.52] | 0.947 |
| PPF | T4 | VL10% - VL30% | 13.28 | [1.31, 25.25] | 0.026 |
| PPF | T4 | VL20% - VL30% | 11.72 | [-0.25, 23.69] | 0.056 |
| PPV | T0 | VL10% - VL20% | 0.01 | [-0.30, 0.32] | 0.994 |
| PPV | T0 | VL10% - VL30% | 0.02 | [-0.29, 0.33] | 0.981 |
| PPV | T0 | VL20% - VL30% | 0.01 | [-0.30, 0.32] | 0.997 |
| PPV | T1 | VL10% - VL20% | 0.03 | [-0.33, 0.40] | 0.972 |
| PPV | T1 | VL10% - VL30% | 0.23 | [-0.13, 0.59] | 0.283 |
| PPV | T1 | VL20% - VL30% | 0.20 | [-0.17, 0.56] | 0.398 |
| PPV | T2 | VL10% - VL20% | 0.02 | [-0.28, 0.33] | 0.983 |
| PPV | T2 | VL10% - VL30% | 0.39 | [0.09, 0.70] | 0.008 |
| PPV | T2 | VL20% - VL30% | 0.37 | [0.07, 0.68] | 0.013 |
| PPV | T3 | VL10% - VL20% | 0.01 | [-0.26, 0.28] | 0.995 |
| PPV | T3 | VL10% - VL30% | 0.25 | [-0.02, 0.52] | 0.075 |
| PPV | T3 | VL20% - VL30% | 0.24 | [-0.03, 0.51] | 0.092 |
| PPV | T4 | VL10% - VL20% | -0.02 | [-0.25, 0.21] | 0.972 |
| PPV | T4 | VL10% - VL30% | -0.00 | [-0.23, 0.23] | 1.000 |
| PPV | T4 | VL20% - VL30% | 0.02 | [-0.21, 0.25] | 0.978 |
| PPP | T0 | VL10% - VL20% | 95.28 | [-221.89, 412.44] | 0.750 |
| PPP | T0 | VL10% - VL30% | 5.94 | [-311.22, 323.11] | 0.999 |
| PPP | T0 | VL20% - VL30% | -89.33 | [-406.50, 227.83] | 0.776 |
| PPP | T1 | VL10% - VL20% | 39.17 | [-312.34, 390.67] | 0.961 |
| PPP | T1 | VL10% - VL30% | 397.06 | [45.55, 748.56] | 0.023 |
| PPP | T1 | VL20% - VL30% | 357.89 | [6.38, 709.40] | 0.045 |
| PPP | T2 | VL10% - VL20% | -37.17 | [-344.33, 269.99] | 0.954 |
| PPP | T2 | VL10% - VL30% | 479.44 | [172.28, 786.60] | 0.001 |
| PPP | T2 | VL20% - VL30% | 516.61 | [209.45, 823.77] | <0.001 |
| PPP | T3 | VL10% - VL20% | 23.39 | [-238.74, 285.51] | 0.975 |
| PPP | T3 | VL10% - VL30% | 299.28 | [37.15, 561.40] | 0.022 |
| PPP | T3 | VL20% - VL30% | 275.89 | [13.76, 538.01] | 0.037 |
| PPP | T4 | VL10% - VL20% | -39.06 | [-270.69, 192.58] | 0.913 |
| PPP | T4 | VL10% - VL30% | 46.56 | [-185.08, 278.19] | 0.879 |
| PPP | T4 | VL20% - VL30% | 85.61 | [-146.03, 317.25] | 0.648 |

Note. Differences are calculated as the first condition minus the second. Confidence intervals and *p* values were Tukey adjusted within each time point. PPF, peak punch force; PPV, peak punch velocity; PPP, estimated peak punch-power index; T0–T4, baseline and 4, 8, 12, and 16 min; CI, confidence interval; VL, velocity loss.
Supplementary Table S6. Tukey-adjusted pairwise comparisons among VL conditions for rear-hand outcomes.

| Outcome | Time | Comparison | Estimated difference | 95% CI | *p* value |
| --- | --- | --- | --- | --- | --- |
| PPF | T0 | VL10% - VL20% | 4.72 | [-18.74, 28.18] | 0.878 |
| PPF | T0 | VL10% - VL30% | 3.39 | [-20.07, 26.85] | 0.935 |
| PPF | T0 | VL20% - VL30% | -1.33 | [-24.79, 22.13] | 0.990 |
| PPF | T1 | VL10% - VL20% | 3.44 | [-8.43, 15.32] | 0.764 |
| PPF | T1 | VL10% - VL30% | 25.39 | [13.52, 37.26] | <0.001 |
| PPF | T1 | VL20% - VL30% | 21.94 | [10.07, 33.82] | <0.001 |
| PPF | T2 | VL10% - VL20% | 2.28 | [-26.57, 31.12] | 0.980 |
| PPF | T2 | VL10% - VL30% | 37.50 | [8.66, 66.34] | 0.008 |
| PPF | T2 | VL20% - VL30% | 35.22 | [6.38, 64.07] | 0.013 |
| PPF | T3 | VL10% - VL20% | 1.44 | [-7.43, 10.32] | 0.919 |
| PPF | T3 | VL10% - VL30% | 4.78 | [-4.10, 13.65] | 0.402 |
| PPF | T3 | VL20% - VL30% | 3.33 | [-5.54, 12.21] | 0.639 |
| PPF | T4 | VL10% - VL20% | -0.44 | [-10.75, 9.86] | 0.994 |
| PPF | T4 | VL10% - VL30% | -3.11 | [-13.42, 7.19] | 0.748 |
| PPF | T4 | VL20% - VL30% | -2.67 | [-12.97, 7.64] | 0.807 |
| PPV | T0 | VL10% - VL20% | -0.04 | [-0.30, 0.21] | 0.912 |
| PPV | T0 | VL10% - VL30% | 0.02 | [-0.23, 0.28] | 0.972 |
| PPV | T0 | VL20% - VL30% | 0.07 | [-0.19, 0.33] | 0.800 |
| PPV | T1 | VL10% - VL20% | 0.01 | [-0.24, 0.27] | 0.992 |
| PPV | T1 | VL10% - VL30% | 0.69 | [0.44, 0.94] | <0.001 |
| PPV | T1 | VL20% - VL30% | 0.68 | [0.43, 0.93] | <0.001 |
| PPV | T2 | VL10% - VL20% | 0.01 | [-0.26, 0.29] | 0.991 |
| PPV | T2 | VL10% - VL30% | 0.65 | [0.38, 0.93] | <0.001 |
| PPV | T2 | VL20% - VL30% | 0.64 | [0.37, 0.91] | <0.001 |
| PPV | T3 | VL10% - VL20% | 0.02 | [-0.21, 0.24] | 0.981 |
| PPV | T3 | VL10% - VL30% | 0.32 | [0.09, 0.54] | 0.004 |
| PPV | T3 | VL20% - VL30% | 0.30 | [0.07, 0.53] | 0.006 |
| PPV | T4 | VL10% - VL20% | 0.01 | [-0.26, 0.28] | 0.993 |
| PPV | T4 | VL10% - VL30% | 0.16 | [-0.11, 0.43] | 0.343 |
| PPV | T4 | VL20% - VL30% | 0.15 | [-0.12, 0.41] | 0.401 |
| PPP | T0 | VL10% - VL20% | -26.44 | [-472.57, 419.68] | 0.989 |
| PPP | T0 | VL10% - VL30% | 43.22 | [-402.91, 489.35] | 0.970 |
| PPP | T0 | VL20% - VL30% | 69.67 | [-376.46, 515.79] | 0.925 |
| PPP | T1 | VL10% - VL20% | 43.61 | [-317.88, 405.10] | 0.954 |
| PPP | T1 | VL10% - VL30% | 1146.83 | [785.34, 1508.32] | <0.001 |
| PPP | T1 | VL20% - VL30% | 1103.22 | [741.73, 1464.71] | <0.001 |
| PPP | T2 | VL10% - VL20% | 71.61 | [-407.57, 550.79] | 0.931 |
| PPP | T2 | VL10% - VL30% | 1281.56 | [802.38, 1760.74] | <0.001 |
| PPP | T2 | VL20% - VL30% | 1209.94 | [730.76, 1689.12] | <0.001 |
| PPP | T3 | VL10% - VL20% | 48.83 | [-291.34, 389.01] | 0.936 |
| PPP | T3 | VL10% - VL30% | 459.67 | [119.49, 799.84] | 0.006 |
| PPP | T3 | VL20% - VL30% | 410.83 | [70.66, 751.01] | 0.014 |
| PPP | T4 | VL10% - VL20% | 12.89 | [-349.01, 374.79] | 0.996 |
| PPP | T4 | VL10% - VL30% | 157.06 | [-204.85, 518.96] | 0.551 |
| PPP | T4 | VL20% - VL30% | 144.17 | [-217.74, 506.07] | 0.604 |

Note. Differences are calculated as the first condition minus the second. Confidence intervals and *p* values were Tukey adjusted within each time point. PPF, peak punch force; PPV, peak punch velocity; PPP, estimated peak punch-power index; T0–T4, baseline and 4, 8, 12, and 16 min; CI, confidence interval; VL, velocity loss.
